# Supplementary material for: Hepatitis C virus NS5A protein promotes the lysosomal degradation of diacylglycerol O-acyltransferase 1 (DGAT1) via endosomal microautophagy
Source: Autophagy Rep. 2022 Jul 22;1(1):264–85. doi: 10.1080/27694127.2022.2095591 (PMC11864691; doi:10.1080/27694127.2022.2095591)
Supplement: Supplemental Material [file KAUO_A_2095591_SM4251.zip › Figure revision_R2_final.pptx]

## Slide 1
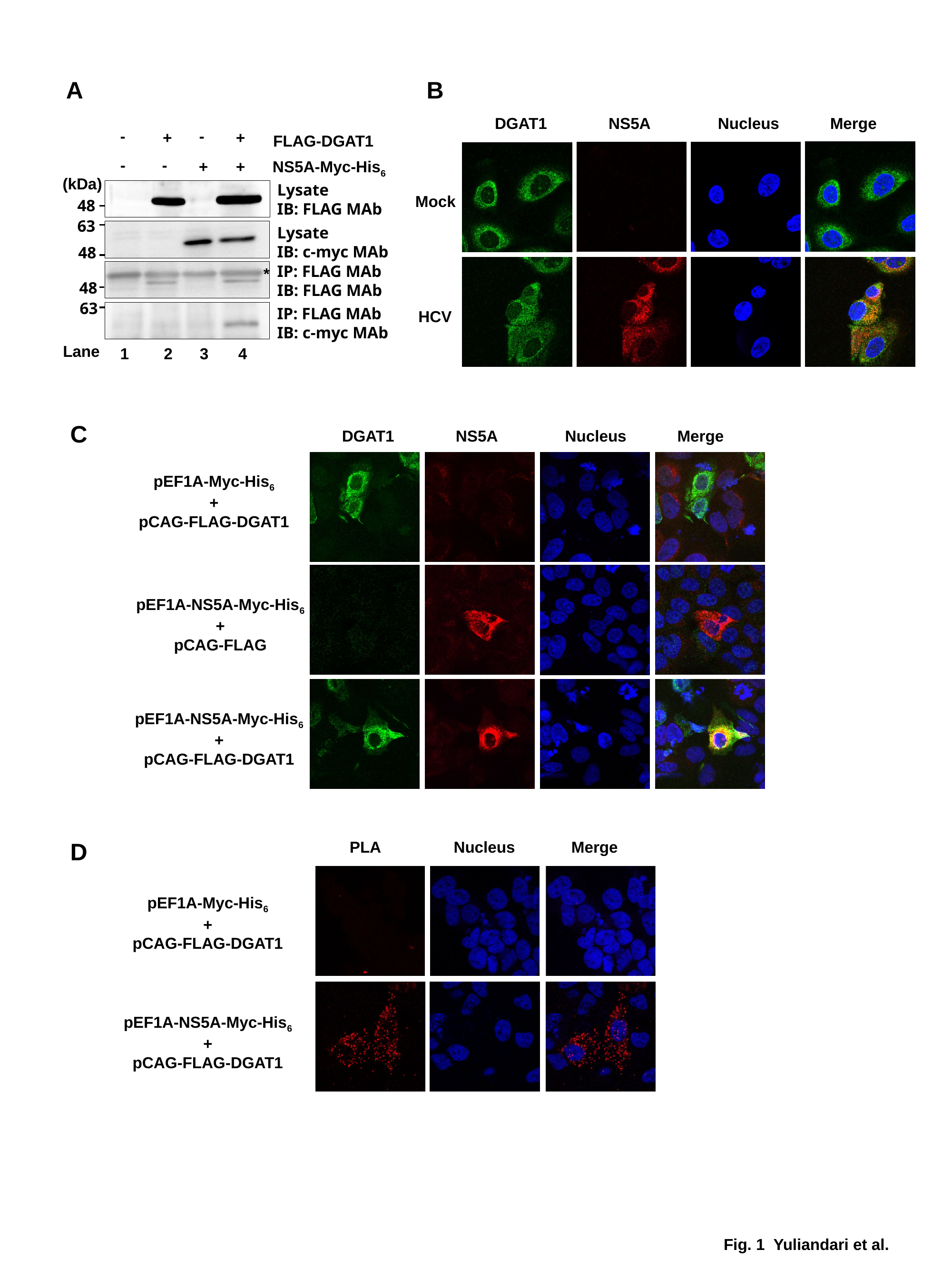

A
B
DGAT1
NS5A
Nucleus
Merge
-
-
+
+
FLAG-DGAT1
-
-
+
+
NS5A-Myc-His6
(kDa)
Lysate
IB: FLAG MAb
48
63
Lysate
IB: c-myc MAb
48
IP: FLAG MAb
IB: FLAG MAb
*
48
63
IP: FLAG MAb
IB: c-myc MAb
Lane
1
2
3
4
Mock
HCV
C
DGAT1
NS5A
Nucleus
Merge
pEF1A-Myc-His6
+
pCAG-FLAG-DGAT1
pEF1A-NS5A-Myc-His6
+
pCAG-FLAG
pEF1A-NS5A-Myc-His6
+
pCAG-FLAG-DGAT1
D
PLA
Nucleus
Merge
pEF1A-Myc-His6
+
pCAG-FLAG-DGAT1
pEF1A-NS5A-Myc-His6
+
pCAG-FLAG-DGAT1
Fig. 1 Yuliandari et al.

## Slide 2
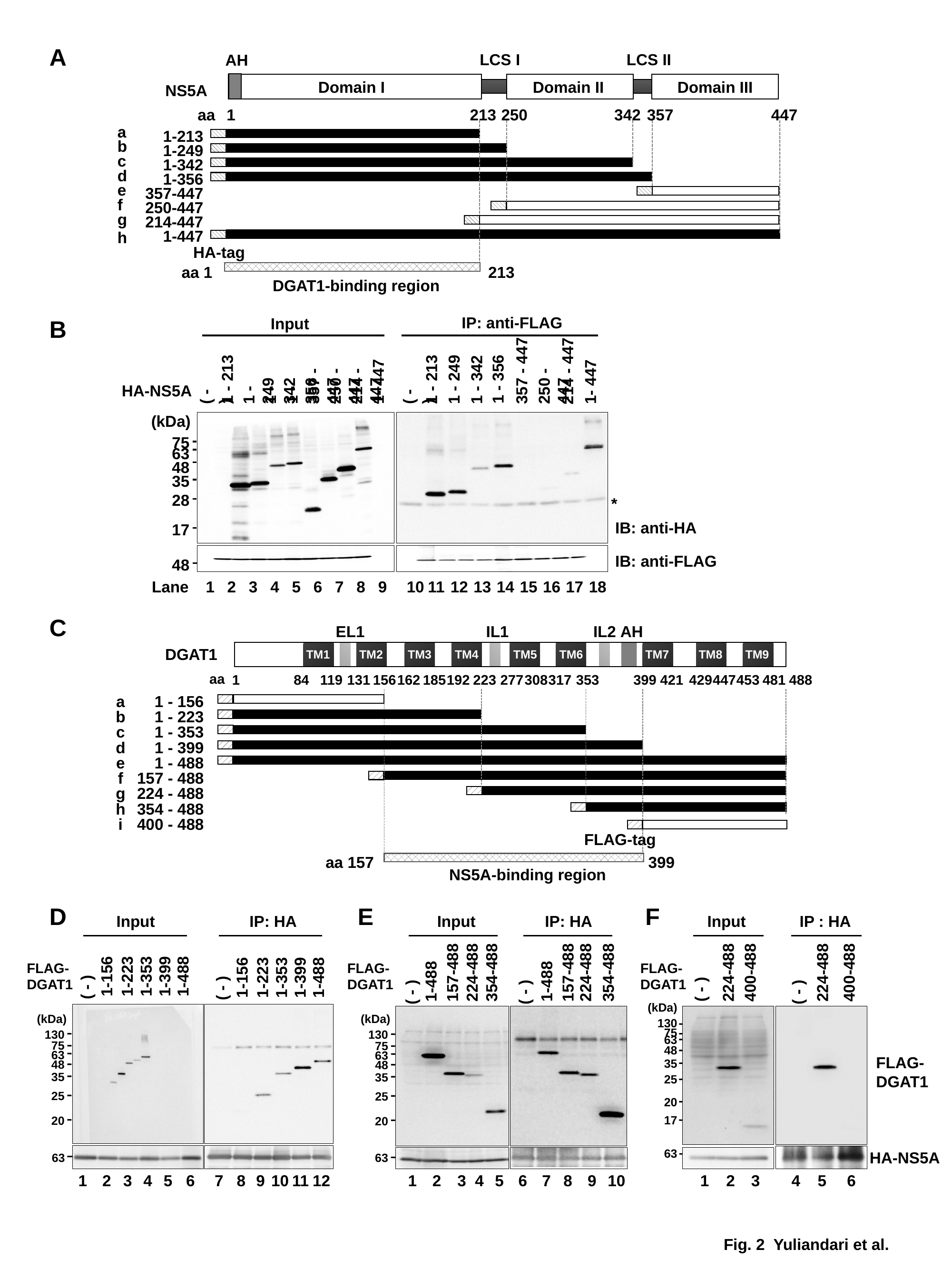

A
LCS I
LCS II
AH
Domain I
Domain II
Domain III
NS5A
aa
1
213
250
342
357
447
a
1-213
b
1-249
c
1-342
d
1-356
e
357-447
f
250-447
g
214-447
1-447
h
HA-tag
aa 1
213
DGAT1-binding region
1 - 356
IP: anti-FLAG
Input
B
214 - 447
357 - 447
1 - 213
1 - 249
1 - 342
250 - 447
214 - 447
1- 447
357 - 447
250 - 447
1- 447
1 - 213
1 - 249
1 - 356
1 - 342
HA-NS5A
( - )
( - )
(kDa)
75
63
48
35
28
*
IB: anti-HA
17
IB: anti-FLAG
48
Lane
1
2
3
4
5
6
7
8
9
10
11
12
13
14
15
16
17
18
C
EL1
IL1
IL2
AH
DGAT1
TM1
TM2
TM3
TM4
TM5
TM6
TM7
TM8
TM9
aa
1
84
119
131
156
162
185
192
223
277
308
317
353
399
421
429
447
453
481
488
1 - 156
a
1 - 223
b
1 - 353
c
1 - 399
d
1 - 488
e
157 - 488
f
224 - 488
g
354 - 488
h
400 - 488
i
FLAG-tag
aa 157
399
NS5A-binding region
D
E
F
Input
IP: HA
Input
IP: HA
Input
IP : HA
FLAG-DGAT1
FLAG-DGAT1
FLAG-DGAT1
224-488
400-488
224-488
400-488
157-488
224-488
354-488
157-488
224-488
354-488
1-488
1-488
1-399
1-488
1-156
1-223
1-353
1-399
1-488
1-156
1-223
1-353
( - )
( - )
( - )
( - )
( - )
( - )
(kDa)
(kDa)
(kDa)
130
75
130
130
63
75
75
48
63
63
FLAG-DGAT1
35
48
48
35
35
25
25
25
20
17
20
20
63
HA-NS5A
63
63
1
2
3
4
5
6
7
8
9
10
11
12
1
2
3
4
5
6
7
8
9
10
1
2
3
4
5
6
Fig. 2 Yuliandari et al.

## Slide 3
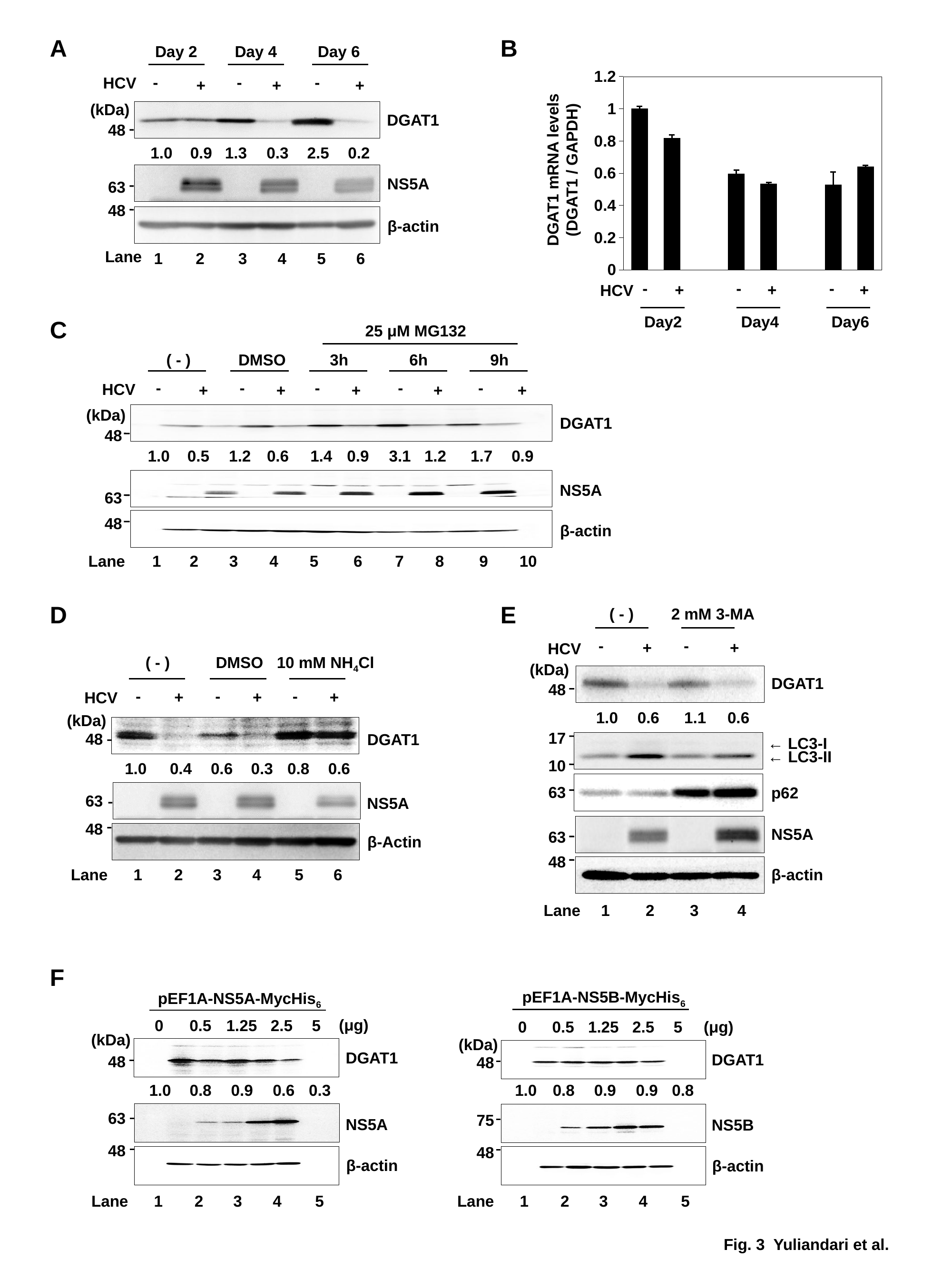

A
B
Day 2
Day 4
Day 6
### Chart
| Category | |
|---|---|-
-
-
HCV
+
+
+
(kDa)
DGAT1
48
1.0
0.9
1.3
0.3
2.5
0.2
DGAT1 mRNA levels
(DGAT1 / GAPDH)
NS5A
63
48
β-actin
Lane
1
2
3
4
5
6
-
-
-
+
+
+
HCV
Day2
Day4
Day6
C
25 μM MG132
( - )
DMSO
3h
6h
9h
-
-
-
-
-
HCV
+
+
+
+
+
(kDa)
DGAT1
48
1.0
0.5
1.2
0.6
1.4
0.9
3.1
1.2
1.7
0.9
NS5A
63
48
β-actin
Lane
1
2
3
4
5
6
7
8
9
10
D
E
( - )
2 mM 3-MA
-
-
+
+
HCV
( - )
DMSO
10 mM NH4Cl
(kDa)
DGAT1
48
-
-
-
+
+
+
HCV
1.0
0.6
1.1
0.6
(kDa)
17
48
DGAT1
← LC3-I
← LC3-II
10
1.0
0.4
0.6
0.3
0.8
0.6
63
p62
63
NS5A
48
NS5A
63
β-Actin
48
β-actin
Lane
1
2
3
4
5
6
4
2
3
Lane
1
F
pEF1A-NS5B-MycHis6
pEF1A-NS5A-MycHis6
(μg)
0
0.5
1.25
2.5
5
(μg)
0
0.5
1.25
2.5
5
(kDa)
(kDa)
DGAT1
DGAT1
48
48
1.0
0.8
0.9
0.6
0.3
1.0
0.8
0.9
0.9
0.8
63
75
NS5A
NS5B
48
48
β-actin
β-actin
Lane
1
2
3
4
5
Lane
1
2
3
4
5
Fig. 3 Yuliandari et al.

## Slide 4
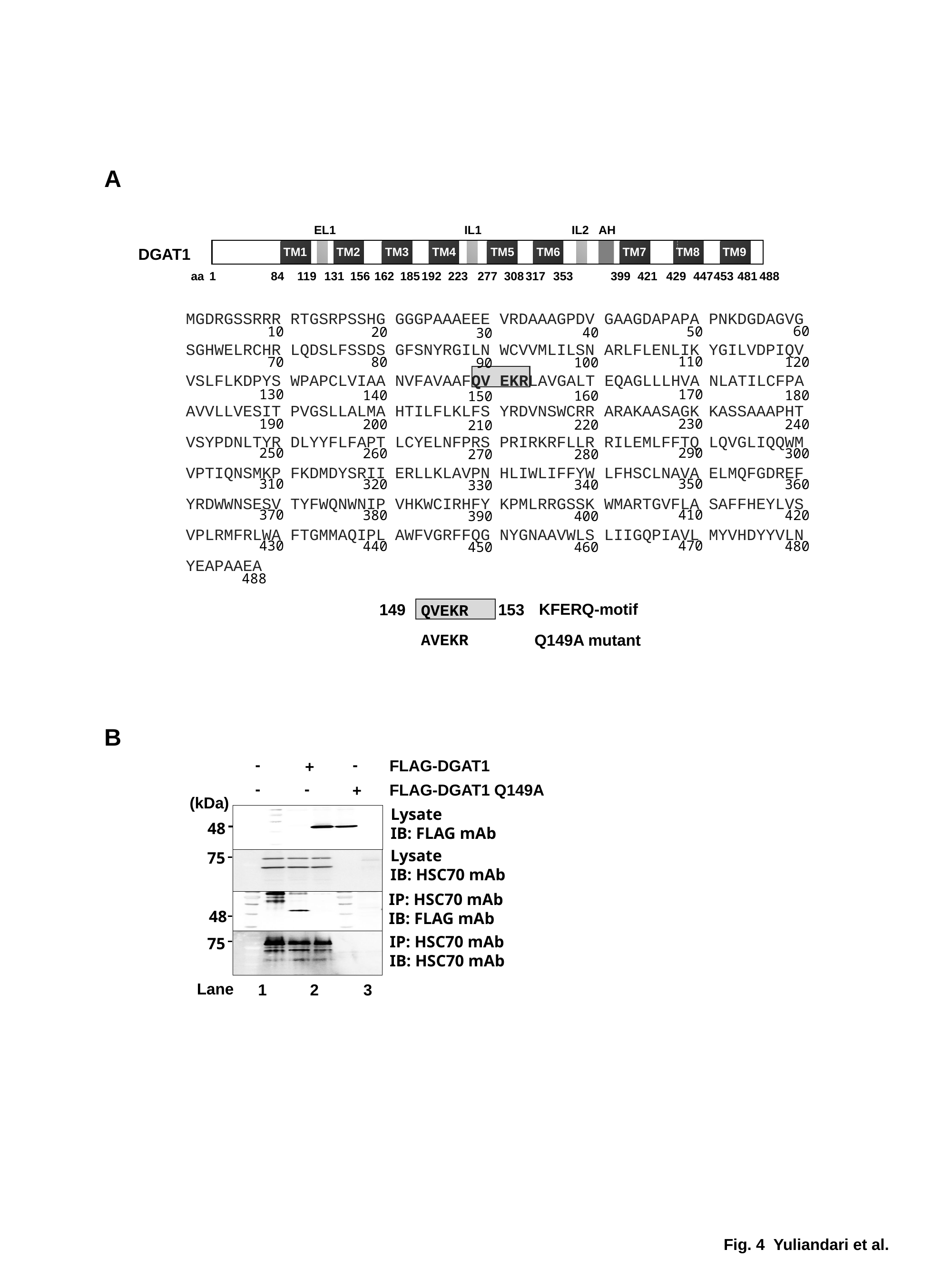

A
EL1
IL1
IL2
AH
DGAT1
TM1
TM2
TM3
TM4
TM5
TM6
TM7
TM8
TM9
aa
1
84
119
131
156
162
185
192
223
277
308
317
353
399
421
429
447
453
481
488
MGDRGSSRRR RTGSRPSSHG GGGPAAAEEE VRDAAAGPDV GAAGDAPAPA PNKDGDAGVG
SGHWELRCHR LQDSLFSSDS GFSNYRGILN WCVVMLILSN ARLFLENLIK YGILVDPIQV
VSLFLKDPYS WPAPCLVIAA NVFAVAAFQV EKRLAVGALT EQAGLLLHVA NLATILCFPA
AVVLLVESIT PVGSLLALMA HTILFLKLFS YRDVNSWCRR ARAKAASAGK KASSAAAPHT
VSYPDNLTYR DLYYFLFAPT LCYELNFPRS PRIRKRFLLR RILEMLFFTQ LQVGLIQQWM
VPTIQNSMKP FKDMDYSRII ERLLKLAVPN HLIWLIFFYW LFHSCLNAVA ELMQFGDREF
YRDWWNSESV TYFWQNWNIP VHKWCIRHFY KPMLRRGSSK WMARTGVFLA SAFFHEYLVS
VPLRMFRLWA FTGMMAQIPL AWFVGRFFQG NYGNAAVWLS LIIGQPIAVL MYVHDYYVLN
YEAPAAEA
60
10
50
20
40
30
110
70
80
120
100
90
130
170
140
180
160
150
190
230
200
240
220
210
250
290
260
300
280
270
310
350
320
360
340
330
370
410
380
420
400
390
430
470
440
480
460
450
488
KFERQ-motif
149
153
QVEKR
AVEKR
Q149A mutant
B
-
-
FLAG-DGAT1
+
-
-
FLAG-DGAT1 Q149A
+
(kDa)
Lysate
IB: FLAG mAb
48
Lysate
IB: HSC70 mAb
75
IP: HSC70 mAb
IB: FLAG mAb
48
IP: HSC70 mAb
IB: HSC70 mAb
75
Lane
1
2
3
Fig. 4 Yuliandari et al.

## Slide 5
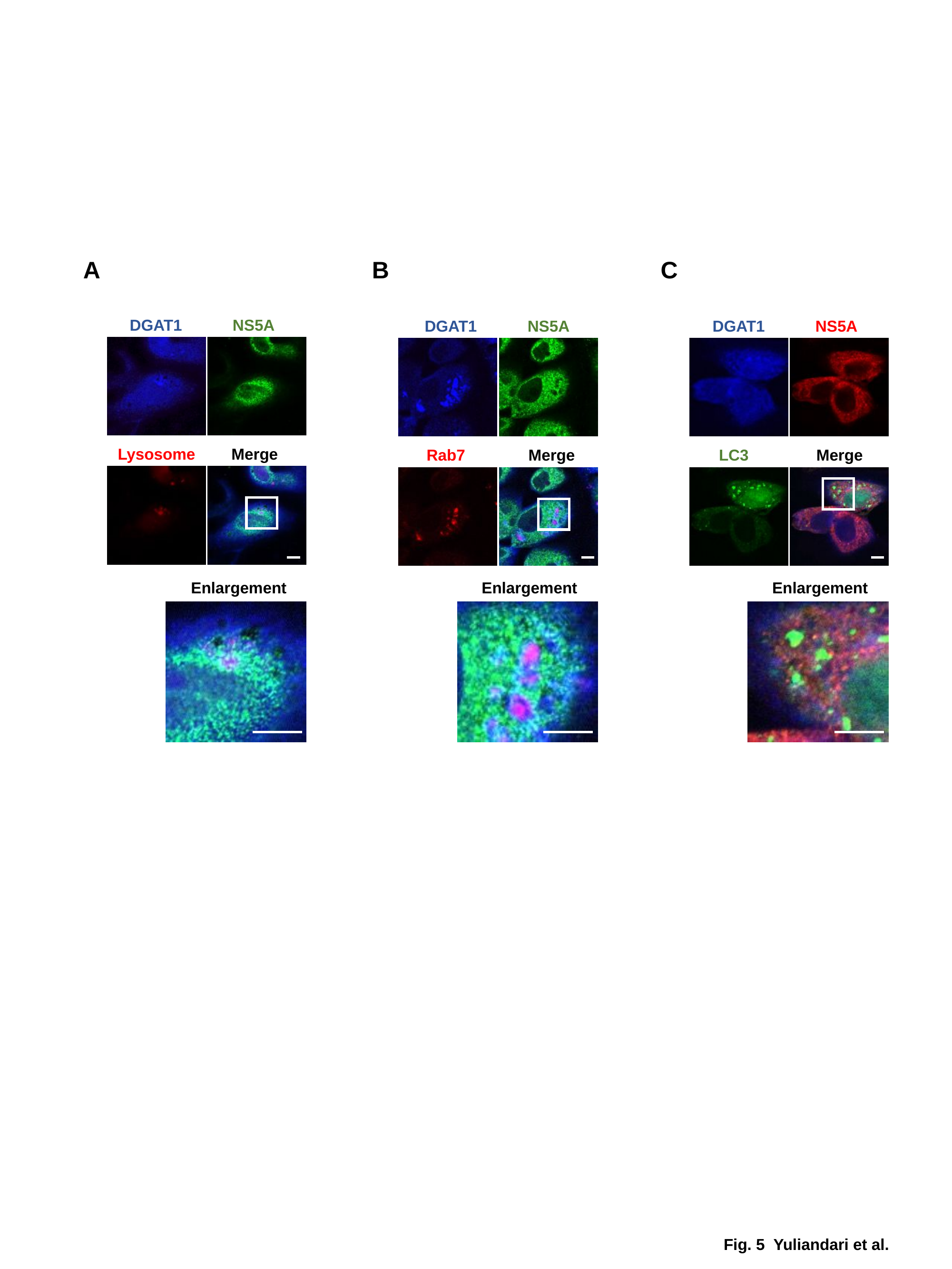

A
B
C
DGAT1
NS5A
DGAT1
NS5A
DGAT1
NS5A
Lysosome
Merge
Rab7
Merge
LC3
Merge
Enlargement
Enlargement
Enlargement
Fig. 5 Yuliandari et al.

## Slide 6
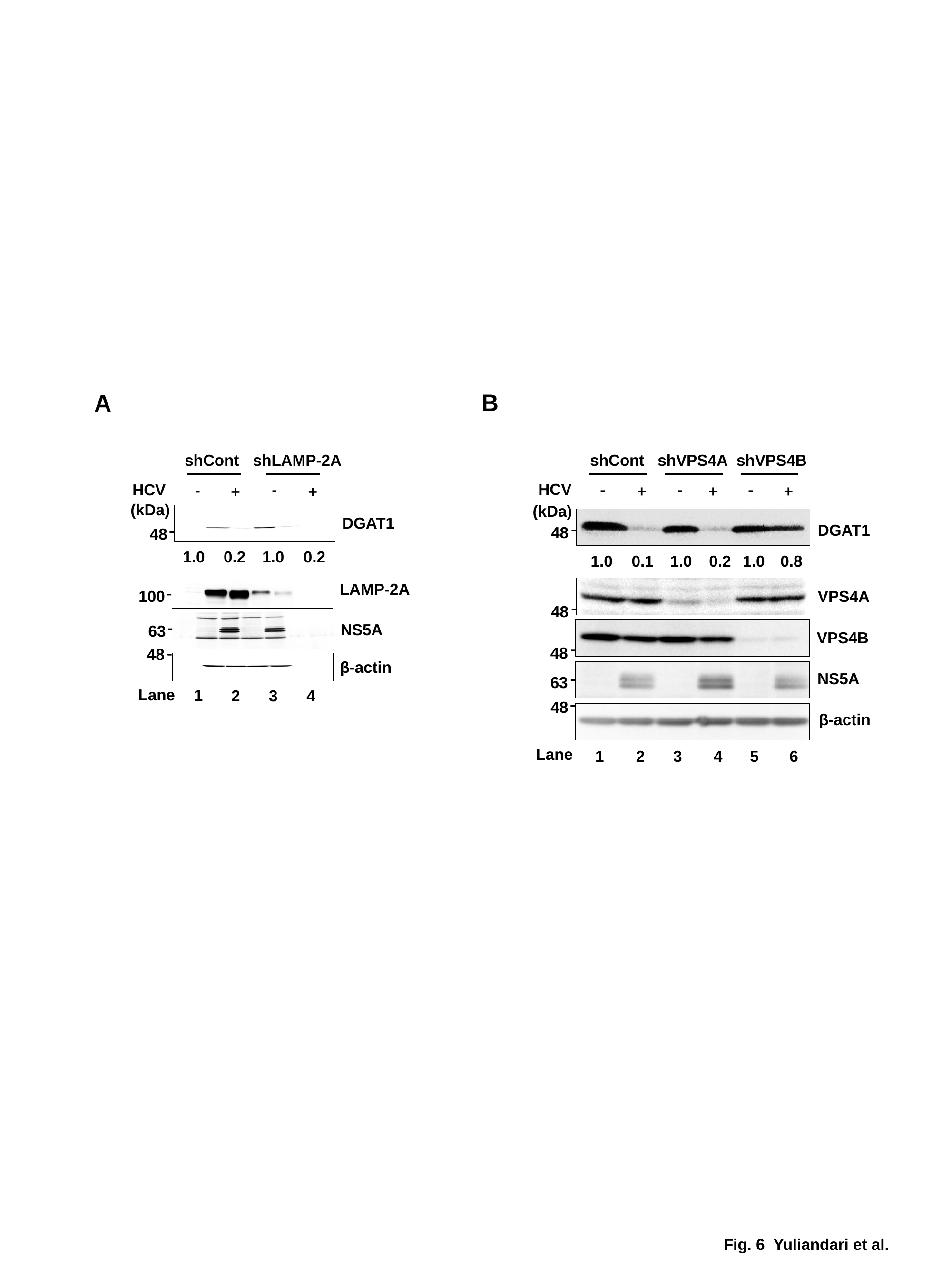

B
A
shCont
shLAMP-2A
shCont
shVPS4A
shVPS4B
HCV
-
-
-
HCV
-
-
+
+
+
+
+
(kDa)
(kDa)
DGAT1
DGAT1
48
48
1.0
0.2
1.0
0.2
1.0
0.1
1.0
0.2
1.0
0.8
LAMP-2A
VPS4A
100
48
NS5A
63
VPS4B
48
48
β-actin
NS5A
63
Lane
1
2
3
4
48
β-actin
Lane
1
2
3
4
5
6
Fig. 6 Yuliandari et al.

## Slide 7
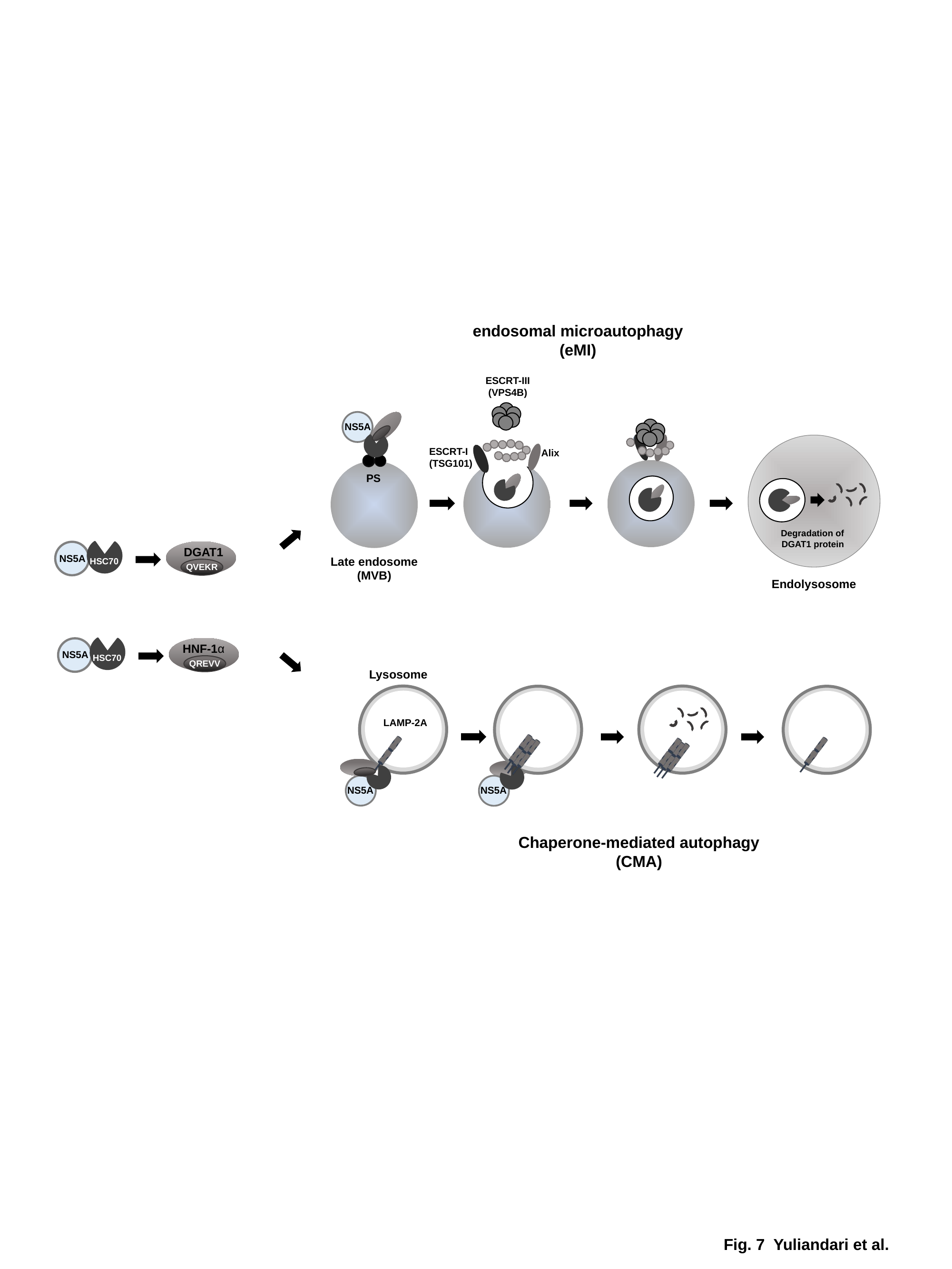

endosomal microautophagy (eMI)
ESCRT-III (VPS4B)
NS5A
ESCRT-I
(TSG101)
Alix
PS
Degradation of DGAT1 protein
Late endosome
(MVB)
Endolysosome
DGAT1
NS5A
HSC70
QVEKR
HNF-1α
NS5A
HSC70
QREVV
Lysosome
LAMP-2A
NS5A
NS5A
Chaperone-mediated autophagy (CMA)
Fig. 7 Yuliandari et al.
