# Supplementary material for: Hepatitis C virus NS5A protein promotes the lysosomal degradation of diacylglycerol O-acyltransferase 1 (DGAT1) via endosomal microautophagy
Source: Autophagy Rep. 2022 Jul 22;1(1):264–85. doi: 10.1080/27694127.2022.2095591 (PMC11864691; doi:10.1080/27694127.2022.2095591)
Supplement: Supplemental Material [file KAUO_A_2095591_SM4251.zip › Yuliandari_Autophagy reports_R2_manuscript tracked changes.pdf]

Research Article KAUO-2022-0039R2

Hepatitis C Virus NS5A Protein Promotes the Lysosomal Degradation of Diacylglycerol O-acyltransferase 1 (DGAT1) via Endosomal Microautophagy

Putu Yuliandari [1, 2], Chieko Matsui [1], Lin Deng [1], Takayuki Abe [1], Hiroyuki Mori [3, 4], Shuhei Taguwa [3, 4], Chikako Ono [3,4], Takasuke Fukuhara [5], Yoshiharu Matsuura [3, 4], Ikuo Shoji [1, #]

[1] Division of Infectious Disease Control, Center for Infectious Diseases, Kobe University Graduate School of Medicine, Kobe, Japan

[2] Department of Clinical Microbiology, Faculty of Medicine, Udayana University, Bali, Indonesia

[3] Laboratory of Virus Control, Center for Infectious Disease Education and Research, Osaka University, Osaka, Japan

[4] Laboratory of Virus Control, Research Institute for Microbial Diseases, Osaka University, Osaka, Japan

[5] Department of Microbiology and Immunology, Faculty of Medicine, Hokkaido University, Sapporo, Hokkaido, Japan

[#]**Correspondence:** Ikuo Shoji, MD, PhD

Professor, Division of Infectious Disease Control, Center for Infectious Diseases, Kobe University Graduate School of Medicine, 7-5-1 Kusunoki-cho, Chuo-ku, Kobe, Japan.

Tel.: +81-78-382-5500; Fax: +81-78-382-5519.

Email: ishoji@med.kobe-u.ac.jp

Short Title: HCV degrades DGAT1 via eMI

ABSTRACT

Many viruses often use a protein degradation system (e.g., the ubiquitin-proteasome pathway or lysosome pathway) to modulate viral propagation and viral pathogenesis. We reported that hepatitis C virus (HCV) infection promotes the lysosomal degradation of hepatocyte nuclear factor-1α (HNF-1α) via chaperone-mediated autophagy (CMA) through an NS5A-mediated association of HNF-1α with cellular chaperone heat shock cognate 70 kDa (HSC70) protein. HSC70 binds to the pentapeptide KFERQ motif (also known as a CMA-targeting motif) on HNF-1α protein and promotes the lysosomal degradation of HNF-1α. The KFERQ motif plays a crucial role in the two lysosomal degradation pathways, CMA and endosomal microautophagy (eMI). Herein, we searched for a novel substrate of HCV-induced lysosomal degradation by examining the NS5A-interacting proteins that carry the KFERQ motif. We identified diacylglycerol O-acyltransferase 1 (DGAT1), which is a key factor for HCV particle formation, as a candidate substrate for HCV-induced lysosomal degradation pathway. The region spanning from amino acids 149–153 of DGAT1 protein matches the rule for the KFERQ motif. DGAT1 protein was co-immunoprecipitated with HSC70, whereas DGAT1 Q149A mutant was not co-immunoprecipitated with HSC70, suggesting that the KFERQ motif is responsible for the interaction between DGAT1 and HSC70. Knockdown of LAMP-2A protein in HCV J6/JFH1-infected cells did not recover DGAT1 protein, whereas knockdown of VPS4B recovered the level of DGAT1 protein, suggesting that DGAT1 is degraded via eMI. These findings lead us to propose that HCV NS5A protein facilitates the recruitment of HSC70 to DGAT1, thereby promoting the lysosomal degradation of DGAT1 via eMI.

Keywords: DGAT1; eMI; endosomal microautophagy; hepatitis C virus; lysosomal degradation; NS5A

Abbreviations: 3-MA: 3-methyladenine; aa: amino acids; AH: amphipathic helix; BSA: bovine serum albumin; CMA: chaperone-mediated autophagy; DAAs: direct-acting antiviral; DGAT1: diacylglycerol O-acyltransferase 1; DMSO: dimethyl sulfoxide; EL: extracellular lumen; eMI: endosomal microautophagy; ESCRT: endosomal sorting complex required for transport; HA: hemagglutinin; HCV: hepatitis C virus; HNF-1α: hepatocyte nuclear factor-1α; HRP: horseradish peroxidase; HSC70: heat shock cognate 70 kDa protein; IB: immunoblotting; IL: intracellular lumen; IP: immunoprecipitation; LAMP-2A: lysosome-associated membrane protein type 2A; LCS: low-complexity sequences; mAb: monoclonal antibody; MOI: multiplicity of infection; MVB: multivesicular bodies; NS: nonstructural protein; pAb: polyclonal antibody; PBS: phosphate-buffered saline; PCR: polymerase chain reaction; PLA: proximity ligation assay; PS: phosphatidylserine; RT: room temperature; TM: transmembrane; TSG: tumor susceptibility gene; VPS4A: vacuolar protein sorting-associated protein 4A; VPS4B: vacuolar protein sorting-associated protein 4B

INTRODUCTION

Approximately 56 million people worldwide are estimated to be infected with hepatitis C virus (HCV), and ~20% of them develop liver cirrhosis or hepatocellular carcinoma [1]. HCV is an enveloped, positive-sense single-stranded RNA virus that belongs to the *Flaviviridae* family, *Hepacivirus* genus. The HCV genome consists of a 9.6-kb RNA encoding a polyprotein of 3,010 amino acids (aa). The polyprotein is cleaved into three structural proteins (core, envelope 1 [E1], and envelope 2 [E2] proteins) and seven nonstructural (NS) proteins (p7, NS2, NS3, NS4A, NS4B, NS5A, NS5B) by viral proteases and host signal peptidases [2]. The structural proteins are involved in the formation of viral particles, whereas the NS proteins participate in viral replication [3]. The development of HCV RNA replicon systems and HCV cell culture systems has enabled us to study HCV replication and the entire HCV-life cycle [4,5]. Recent advances in HCV research have resulted in the development of novel anti-HCV therapeutics, i.e., direct-acting antivirals (DAAs), which have dramatically improved the treatment of chronic hepatitis C. However, the emergence of resistance-associated substitutions raises new concerns [6].

We reported that HCV infection promotes the lysosomal degradation of hepatocyte nuclear factor-1α (HNF-1α) via chaperone-mediated autophagy (CMA) through an NS5A-mediated association of HNF-1α with cellular chaperone heat shock cognate 70 kDa (HSC70) protein; we demonstrated that lysosome-associated membrane protein type 2A (LAMP-2A) is required for the degradation of HNF-1α [7]. However, little is known about the roles of HCV-induced lysosomal degradation in the HCV life cycle and viral pathogenesis. In this study, we searched for a novel substrate of an HCV-induced

lysosomal degradation pathway by examining the NS5A-interacting proteins that carry the KFERQ motif, which is important for the association with HSC70.

Autophagy is a cellular process that transports materials from the cytoplasm to the lysosome for degradation. This process contributes to cells' survival by removing damaged organelles and protein aggregates and promoting cellular homeostasis [8]. There are at least three types of autophagy in mammalian cells: macroautophagy, microautophagy, and chaperone-mediated autophagy (CMA). In microautophagy, cytosolic components are directly ingested by lysosomes through an invagination of the lysosomal membrane. One of the selective forms of microautophagy is endosomal microautophagy (eMI). Both eMI and CMA utilize the cellular chaperone protein HSC70 to selectively recognize substrate proteins with a KFERQ motif [9-11].

The selectivity of the target protein is determined by the presence of a specific pentapeptide motif, the KFERQ motif, in the amino acid sequence of the substrate protein [12-14]. The KFERQ motif contains one or two of the positively charged residues lysine (K) and arginine (R); one or two of the hydrophobic residues phenylalanine (F), isoleucine (I), leucine (L), and valine (V); one of the negatively charged residues aspartic acid (D) or glutamic acid (E); and one glutamine (Q) on either side of the pentapeptide [15-17]. Cuervo's group developed free Web-based software, KFERQ finder V0.8 (https://rshine.einsteinmed.org/), to quickly identify this motif in any protein sequence [18].

The interaction between HSC70 and the target protein is necessary for two selective lysosomal autophagy pathways: CMA and eMI. In CMA, the protein complex formed by HSC70 interacts with LAMP-2A, causing the target protein to unfold and degrade in the lysosome. After the substrate transfer to LAMP-2A, HSC70 is released

into the cytosol. In contrast to CMA, eMI does not require the unfolding or binding of cytosolic proteins to LAMP-2A. After binding to a substrate protein, HSC70 interacts with phosphatidylserine (PS) of the endosomal membrane. A substrate protein for eMI is sequestered by the formation of an invagination in the surface of the endosomal membrane, which is mediated by tumor susceptibility gene (TSG) 101 as an endosomal sorting complex required for transport (ESCRT I) and three proteins: vacuolar protein sorting-associated protein (VPS) 4A, VPS4B, and Alix [19-21].

Diacylglycerol O-acyltransferase 1 (DGAT1) is an important enzyme in the final step of triglyceride synthesis. DGAT1 plays a crucial role in HCV infection by recruiting the HCV core protein onto the surface of cellular lipid droplets [22,23]. In this study, we observed that DGAT1 is degraded via the lysosomal degradation pathway. We aim to clarify the molecular mechanisms underlying the lysosomal degradation of DGAT1 protein induced by HCV NS5A protein. Here, we demonstrate that HCV NS5A protein promotes the lysosomal degradation of DGAT1 protein via eMI, but not CMA.

RESULTS

HCV NS5A protein interacts with DGAT1.

To investigate whether HCV NS5A protein interacts with DGAT1 protein in Huh-7.5 cells, we cotransfected pCAG-FLAG-DGAT1 together with pEF1A-NS5A-Myc-His_6 into Huh-7.5 cells. The immunoprecipitation analysis revealed that with the use of anti-FLAG mAb, NS5A-Myc-His_6 was coimmunoprecipitated with FLAG-DGAT1 protein (Fig. 1A, bottom panel, lane 4). These results suggest that HCV NS5A protein interacts with DGAT1 protein in Huh-7.5 cells. To examine the subcellular localization of NS5A and DGAT1, we performed immunofluorescence staining; it demonstrated that NS5A

protein was colocalized with endogenous DGAT1 in the cytoplasm in HCV J6/JFH1-infected cells (Fig. 1B, lower panel, merge).

To examine the subcellular localization of NS5A and DGAT1 in Huh-7.5 cells, the cells were cotransfected with pCAG-FLAG-DGAT1 and pEF1A-NS5A-Myc-His_6. Immunofluorescence staining revealed that FLAG-DGAT1 was colocalized with NS5A-Myc-His_6 in the cytoplasm (Fig. 1C, bottom panel, merge). To further examine whether DGAT1 is colocalized with NS5A protein, we performed a proximity ligation assay (PLA). The PLA revealed a strong signal in the presence of both pCAG-FLAG-DGAT1 and pEF1-NS5A-Myc-His_6 (Fig. 1D, bottom panel, merge). These results suggest that NS5A interacts with DGAT1 in Huh-7.5 cells.

NS5A domain I binds to DGAT1 protein.

To map the DGAT1-binding region on NS5A protein, we performed a coimmunoprecipitation analysis using a series of hemagglutinin (HA)-tagged NS5A deletion mutants (Fig. 2A). All of the HA-NS5A proteins except HA-NS5A (357-447), HA-NS5A (250-447) and HA-NS5A (214-447) (Fig. 2B, upper panel, lanes 15–17) were coimmunoprecipitated with FLAG-DGAT1 protein with the use of anti-FLAG mAb. These results suggest that NS5A domain I consisting of aa 1 to 213 is important for DGAT1 binding.

We further mapped the NS5A-binding region on DGAT1 protein using a series of FLAG-tagged DGAT1 deletion mutants (Fig. 2C). All of the FLAG-DGAT1 deletion mutants except FLAG-DGAT1 (aa 1–156) and FLAG-DGAT1 (aa 400–488) (Fig. 2D, upper panel, lane 8; Fig. 2F, upper panel, lane 6) were coimmunoprecipitated with NS5A-

Myc-His_6 with the use of anti-HA pAb. These results suggest that the region from aa 157 to 399 on DGAT1 is important for the interaction with NS5A protein.

HCV NS5A is important for the lysosomal degradation of DGAT1 protein.

To determine whether HCV infection promotes degradation of DGAT1 protein, we examined the endogenous DGAT1 protein levels by performing an immunoblot analysis in HCV J6/JFH1-infected Huh-7.5 cells. The DGAT1 protein levels were decreased at day 4 and day 6 post-infection in HCV J6/JFH1-infected cells (Fig. 3A, upper panel, lanes 4 and 6). However, there was no significant difference between DGAT1 mRNA level in HCV-uninfected cells and that in the HCV-infected cells (Fig. 3B). These results suggest that HCV-induced DGAT1 protein reduction is not due to transcriptional repression.

Next, to determine whether protein degradation is involved in the HCV-induced reduction of DGAT1 protein, we assessed the potential role of proteasomal or lysosomal protease on DGAT1 protein. We treated the cells with a proteasome inhibitor, MG132, or a lysosomal protease inhibitor, ammonium chloride (NH_4Cl). MG132 did not increase the levels of DGAT1 protein (Fig. 3C, upper panel, lanes 6, 8 and 10), whereas NH_4Cl restored the levels of DGAT1 protein (Fig. 3D, upper panel, lane 6). These results suggest that HCV infection induces the lysosomal degradation of DGAT1 protein.

To determine whether macroautophagy is involved in HCV-induced reduction of DGAT1 protein, we treated the cells with a macroautophagy inhibitor, 3-methyladenine (3-MA). Inhibition of macroautophagy, as evidenced by the accumulation of SQSTM1/p62 and a reduction in LC3-II form (Fig. 3E, 2nd and 3rd panels, lane 4), did

not increase DGAT1 protein levels (Fig. 3E, upper panel, lane 4). This result suggests that macroautophagy is not involved in the HCV-induced reduction of DGAT1 protein.

For the investigation of a possible role of NS5A in the degradation of the endogenous DGAT1 protein, Huh-7.5 cells were transfected with increasing amounts of either pEF1A-NS5A-Myc-His_6 or pEF1A-NS5B-Myc-His_6. Overexpression of NS5A-Myc-His_6, but not NS5B-Myc-His_6, significantly reduced DGAT1 protein (Fig. 3F, upper panels). These results indicate that NS5A protein specifically reduces endogenous DGAT1 protein.

The KFERQ motif of DGAT1 is required for the interaction with HSC70.

To determine whether DGAT1 contains a KFERQ motif, we analyzed the amino acid sequence of DGAT1 based on the rules for the consensus sequence of the KFERQ motif [14]. Following this rule, we observed a putative KFERQ motif in the region spanning from aa 149 to 153 on DGAT1 protein (Fig. 4A). The pentapeptide ^{149}QVEKR^{153} completely matches the rules for the KFERQ motif. To determine whether the putative KFERQ motif on DGAT1 protein is required for the interaction with HSC70, we constructed pCAG-FLAG-DGAT1 Q149A (encoding a substitution of Q to A at the position of aa 149). The results of the coimmunoprecipitation analysis demonstrated that endogenous HSC70 interacted with FLAG-DGAT1 (Fig. 4B, third panel, lane 2) but not with FLAG-DGAT1 Q149A (Fig. 4B, third panel, lane 3). These results suggest that HSC70 interacts with DGAT1 via the KFERQ motif.

HCV NS5A is colocalized with DGAT1 in the late endosome and the lysosome.

To examine the subcellular colocalization of DGAT1 and NS5A in HCV-infected Huh-7.5 cells, we performed immunofluorescence staining. We used LysoTracker as a marker for lysosome, Rab7 as a marker for late endosome, and LC3 as a marker of autophagosome. We found merged white signals in the lysosome (Fig. 5A, merge, enlarged image) and the late endosome (Fig. 5B, merge, enlarged image). In contrast, merged white signals were not detected in the autophagosome (Fig. 5C, merge, enlarged image). These results suggest that DGAT1 and NS5A protein are colocalized in the late endosome and the lysosome.

HCV-induced degradation of DGAT1 protein was restored by knockdown of VPS4B but not LAMP-2A.

The interaction of cellular chaperone HSC70 with a host protein via the KFERQ motif is important in two selective lysosomal degradation pathways, CMA and eMI. To determine whether DGAT1 is degraded via CMA or eMI, we performed knockdown experiments using short hairpin (sh)RNA. To determine whether LAMP-2A, a specific receptor for the CMA pathway, plays a role in the HCV-induced degradation of DGAT1 protein, we made stable LAMP-2A knockdown Huh-7.5 cells (shLAMP-2A Huh-7.5 cells). The endogenous DGAT1 protein level was not recovered in HCV-infected shLAMP-2A Huh-7.5 cells (Fig. 6A, upper panel, lane 4). These results suggest that the CMA pathway is not involved in the HCV-induced lysosomal degradation of DGAT1 protein.

To investigate whether HCV induces DGAT1 protein degradation via eMI, we made stable VPS4A-knockdown cells and VPS4B-knockdown cells using shRNA. DGAT1 protein was recovered in shVPS4B-Huh-7.5 cells (Fig. 6B, upper panel, lane 6).

On the other hand, DGAT1 protein was not recovered in VPS4A- Huh-7.5 cells (Fig. 6B, upper panel, lane 4). These results suggest that HCV induces the lysosomal degradation of DGAT1 protein via eMI and that VPS4B plays an important role in eMI.

Taking these findings together, we propose that HCV infection promotes the lysosomal degradation of DGAT1 protein via eMI (Fig.7).

DISCUSSION

We have reported that HCV NS5A protein promotes the lysosomal degradation of HNF-1α via CMA through an NS5A-mediated association of HNF-1α with cellular chaperone HSC70 protein [7,24,25]. To seek a novel target of HCV-induced CMA, we focused on DGAT1 among the NS5A-interacting proteins. However, to our surprise, we discovered that HCV NS5A protein promotes the lysosomal degradation of DGAT1 via eMI, but not CMA, using NS5A, HSC70, and KFERQ motif on DGAT1.

The immunoprecipitation analysis revealed that DGAT1 protein was co-immunoprecipitated with HCV NS5A protein. The immunofluorescent staining and the results of the PLA suggest that NS5A protein interacted with DGAT1 protein in Huh-7.5 cells (Fig. 1). We mapped the DGAT1-binding domain on NS5A protein and found that the region spanning from aa 1 to aa 213 is important for the interaction with DGAT1. We also mapped the NS5A-binding domain on DGAT1 protein and found that the region spanning from aa 157 to aa 399 is important for the interaction with NS5A (Fig. 2). Using the lysosomal protease inhibitor NH_4Cl, we demonstrated that DGAT1 is degraded via an NS5A-dependent lysosomal degradation pathway. Treatment with 3-MA did not recover DGAT1 protein level, suggesting that macroautophagy is not involved in the reduction of DGAT1 protein (Fig. 3).

We demonstrated that DGAT1 protein contains a KFERQ motif in the region spanning from aa 149 to aa 153. The immunoprecipitation analysis revealed that the KFERQ motif is crucial for the interaction between DGAT1 and HSC70 (Fig. 4). Immunofluorescence staining showed that DGAT1 protein and NS5A protein was colocalized in the late endosome and the lysosome (Fig. 5). However, the HCV-induced degradation of DGAT1 protein was restored in shVPS4B Huh-7.5 cells, but not in shLAMP-2A Huh-7.5 cells (Fig. 6). Taken together, these results suggest that HCV NS5A protein interacts with HSC70 and DGAT1, thereby promoting the lysosomal degradation of DGAT1 via eMI in a VPS4B-dependent manner (Fig. 7). To our knowledge, this is the first report clarifying the molecular mechanism of eMI induced by HCV infection.

HCV NS5A is a large phosphoprotein (56–58 kDa) organized into three domains. NS5A domain I is essential for HCV RNA replication and is relatively conserved among HCV genotypes compared to domains II and III, suggesting that an NS5A-DGAT1 interaction is common to all HCV genotypes [26,27]. With the use of a series of NS5A deletion mutants (Fig. 2A), our findings demonstrated that domain I of NS5A is important for the association with DGAT1 protein (Fig. 2B). DGAT1 is an integral membrane protein synthesizing triacylglycerides from diacylglycerol and fatty acyl-CoA. DGAT1 has nine transmembrane helices, TM1–TM9, and three long loops [26]. With a series of DGAT1 deletion mutants (Fig. 2C), the results demonstrated that the region spanning from aa 157 to aa 399 of DGAT1 protein is important for the association with the NS5A protein (Fig. 2D–F).

The pentapeptide ^{149}QVEKR^{153} of DGAT1 completely matches the rules for the KFERQ motif. A coimmunoprecipitation result showed that endogenous HSC70

interacted with wildtype DGAT1 protein, but not with DGAT1 Q149A mutant (Fig. 4B), emphasizing the importance of the KFERQ motif in target protein for the interaction with HSC70. We reported that the region spanning from aa 1 to aa 126 of NS5A protein is important for the interaction with HSC70 [7].

In eMI, substrate proteins in vesicles are degraded in the late endosome or through the fusion with the lysosomes (endolysosome) [20, 21]. Therefore, the HCV NS5A may be colocalized with DGAT1 protein in both the endosome and the lysosome. It is still unclear whether the entire ESCRT machinery is necessary for the eMI pathway [19-21]. The substrate protein's intrinsic properties may be responsible for switching between CMA and eMI pathways. Because these two pathways require distinct receptors to transport the target protein to the lysosome, knocking down each receptor aids in the analysis of protein degradation; for example, knocking down the LAMP-2A membrane protein increases the amount of target protein in the CMA pathway. On the other hand, when the VPS4A/B protein is knocked down, the substrate protein level increases in the eMI pathway [14,21]. In our present study, the DGAT1 protein expression level was not recovered in HCV-infected shLAMP-2A cells, suggesting that the CMA pathway is not involved in the degradation of DGAT1 protein. The DGAT1 protein expression level was recovered only in shVPS4B-Huh-7.5 cells, suggesting that HCV NS5A induced the lysosomal degradation of DGAT1 protein via eMI in a VPS4B-dependent manner. However, further experiments are needed to understand how substrate specificity is determined between CMA and eMI.

DGAT1 interacts with HCV core protein and is required for the trafficking of the core to lipid droplets [22]. DGAT1 localizes NS5A protein to lipid droplets and enhances the NS5A-core interaction to promote the production of viral particles [23]. Our present

findings provide evidence suggesting that HCV promotes the lysosomal degradation of DGAT1 protein via eMI. HCV infection paradoxically downregulates DGAT1 protein in the cells. We are currently seeking to determine the physiological significance of HCV-induced DGAT1 degradation in HCV life cycle. In our preliminary result, we observed that overexpression of DGAT1 protein resulted in decrease of extracellular HCV infectivity titers, although there were no significant differences in intracellular and extracellular HCV RNA levels and intracellular HCV infectivity titers between control cells and DGAT1-overexpressed cells (data not shown). We speculate that removal of too much DGAT1 protein or quality control of DGAT1 protein via eMI may be important for efficient production of infectious HCV particles. Further research is necessary to elucidate a pathophysiological role of the HCV-induced lysosomal degradation of DGAT1 protein via eMI.

DGAT1 plays an important role in the final step of triglyceride synthesis. Human DGAT1 is expressed primarily in the small intestine and liver. A study using DGAT1-deficient Huh-7.5 cell lines demonstrated dedifferentiated and stem cell-like characteristics in stem cell culture medium without serum. The complete and long-term silencing of DGAT1 decreased E-cadherin and integrin β1, an adhesion molecule that contributes to the cell-extracellular matrix or cell-substrate adhesion, similar to the process of liver cirrhosis without fatty degeneration [28,29]. It may be informative to investigate the pathogenesis of HCV-induced DGAT1 downregulation in terms of liver cirrhosis.

In conclusion, we propose that HCV NS5A interacts with HSC70 and recruits HSC70 to DGAT1, thereby promoting the lysosomal degradation of DGAT1 via eMI.

Further investigations of the HCV-induced selective degradation of host proteins via CMA and eMI may contribute to our understanding of the pathogenesis of HCV.

MATERIALS AND METHODS

Cell culture and viruses. A human hepatoma cell line, Huh-7.5 cells, was provided by Dr. Charles M. Rice (The Rockefeller University, New York, NY)[30]. The cells were cultured in Dulbecco's modified Eagle's medium (DMEM) (high glucose) with L-glutamine and phenol red (Fuji Film Wako Pure Chemical Industries, Ltd., 044-29765) and supplemented with 50 IU/ml penicillin, 50 μg/ml streptomycin (Gibco, 15-140-122), 10% heat-inactivated fetal bovine serum (Biowest, S1760-500), and 0.1 mM nonessential amino acids (Invitrogen, 11140050) at 37°C in a 5% CO_2 incubator. Cells were washed using PBS (-) solution (Nissui Pharmaceutical Co., Ltd., 05913). Cells were transfected with plasmid DNA using FuGENE 6 transfection reagents (Promega, E269A). The pFL-J6/JFH1 plasmid, which encodes the entire viral genome of a chimeric strain of HCV-2a, J6/JFH1 was provided by Dr. C.M. Rice [5]. The HCV genome RNA was synthesized *in vitro* using pFL-J6/JFH1 as a template and was transfected into Huh-7.5 cells by electroporation [4,5,31,32]. The virus produced in the culture supernatant was used for infection experiments [31].

Expression plasmids. The expression plasmids for NS5A and a series of NS5A deletion mutants constructed as HA-tagged or Myc-His_6-tagged proteins have been described [7,24,25,33]. The expression plasmids pEF-FLAG-NS5A [34], pDsRed-Monomer-Rab7-C3 [35], and pLEF-GFP-LC3 [36] were kindly provided by Dr. Y. Matsuura (Osaka University, Osaka, Japan). The expression plasmid for HSC70 has been described [7].

The cDNA fragment of DGAT1 was amplified by PCR using pCMV6-XL4-DGAT1 as a template. The specific primers used for PCR were as follows: sense primer 5'-TCGAGCTCAGCGGCCATGGGCGACCGCGGCAGC-3' and anti-sense primer 5'-AGTGAATTCGCGGCCTCAGGCCTCTGCCGCTGG-3'.

The amplified PCR product was purified and inserted into NotI site of pCAG-FLAG using an In-Fusion HD cloning kit (Takara Bio USA, Inc., 639649). The Q149A point mutant of DGAT1 was constructed by overlap extension PCR using pCAG-FLAG-DGAT1 as a template. The specific primer used for PCR were as follows: sense primer (Q149A) 5'-GTGGCTGCATTCCGGGTTGAGAAGCGCCTG-3' and anti-sense primer (Q149A), 5'CAGGCGCTTCTCAACCCGGAATGCAGCCAC-3'. The sequences of the inserts were extensively verified by sequencing (Eurofins Genomics, Tokyo).

Antibodies and reagents. The mouse monoclonal antibodies (mAbs) used in this study were anti-FLAG (M2) mAb (Sigma-Aldrich, F-3165), anti-HSC70 (B-6) mAb (Santa Cruz Biotechnology, sc-7298), anti-c-Myc (9E10) mAb (Santa Cruz Biotechnology, sc-40), anti-ß-actin mAb (Sigma-Aldrich, A-5441), anti-DDDDK-tag mAb (MBL International Corporation, M185-3L), anti-VPS4B (A-11) mAb (Santa Cruz Biotechnology, sc-377162) and anti DGAT1 (A-5) mAb (Santa Cruz Biotechnology, sc-271934).

The rabbit polyclonal antibodies (pAbs) used in this study were anti-HA pAb (Sigma-Aldrich, H-6908), anti-LAMP2A pAb (Abcam, ab18528), anti-DDDDK-tag pAb (MBL International Corporation, PM020), anti-VPS4A (UT289) pAb (Millipore, ABS1646), anti-NS5A (2914-1) pAb (a kind gift from T. Wakita, National Institute of Infectious Diseases, Tokyo), anti-LC3 (PM036) pAb (MBL Life Science, PM036), and

anti-p62 (Cell Signaling Technology, 5114). Horseradish peroxidase (HRP)-conjugated anti-mouse IgG (Cell Signaling Technology, 7076S) and HRP-conjugated anti-rabbit IgG (Cell Signaling Technology, 7074S) were used as secondary antibodies. MG132 was purchased from Fuji Film Wako Pure Chemical Industries, Ltd., 135-18453. Ammonium chloride (NH_4Cl) was purchased from Fuji Film Wako Pure Chemical Industries, Ltd., 017-02995. The macroautophagy inhibitor 3-MA was purchased from Sigma-Aldrich Co., LLC., M9281.

Immunoblot analysis. The immunoblot (IB) analysis was performed essentially as described [24,37-39]. The cell lysates were separated by 10% or 15% sodium dodecyl sulfate-polyacrylamide gel electrophoresis (SDS-PAGE) and transferred to a 0.45 μm Immobilon-P polyvinylidene difluoride membrane (PVDF) (Millipore, IPVH00010). The membranes were incubated with a primary antibody, followed by incubation with a peroxidase-conjugated secondary antibody. The positive bands were visualized using Amersham enhanced chemiluminescence (ECL) western blotting detection reagents (Cytiva, RPN2106). The intensity of bands was quantified using ImageJ 1.53r software.

Immunoprecipitation. Cultured cells were lysed with a buffer containing 150 mM NaCl, 50 mM Tris-HCl (pH 7.5), 1% NP-40, 1 mM EDTA, 100% glycerol, and cOmplete™, EDTA-free, protease inhibitor cocktail (Roche Diagnostics, 05056489001). The lysate was centrifuged at 12,500 *g* for 15 min at 4°C, and the supernatant was immunoprecipitated with appropriate antibodies. Immunoprecipitation (IP) was performed as described [7,37]. Briefly, the cell lysates were immunoprecipitated with anti-FLAG M2 affinity gel (Sigma-Aldrich Co., A2220) or Protein A-Sepharose 4 Fast

Flow (GE Healthcare, GE17-5280-04) incubated with appropriate antibodies at 4°C for 4 h. After being washed with the lysis buffer five times, the immunoprecipitants were analyzed by immunoblotting.

RNA interference and stable knockdown cells. The short hairpin RNA (shRNA) target sequences for LAMP-2A, VPS4A, VPS4B, and scramble were as follows:
shLAMP-2A, 5'- GGCAGGAGUACUUAUUCUA-3',
shVPS4A, 5'-GCUGAAGGAUUAUUUACGA-3',
shVPS4B, 5'-AGCGAUAGAUCUGGCUAGCAA-3',
and shScramble, 5'-GGACAUCGACGGCUUUAUA-3'. The shVPS4A and shVPS4B were inserted into the pSilencer 2.1 U6 puro vector (Ambion, AM5762). The shLAMP2A was inserted into the pSilencer 2.1 U6 hygro vector (Ambion, AM5760). Huh-7.5 cells were transfected with the plasmids and drug-resistant clones were selected by 1 μg/ml puromycin (Sigma-Aldrich, P9620) or 200 μg/ml hygromycin B (Nacalai Tesque, 09287-84) to establish the stable knockdown cells for LAMP-2A, VPS4A, and VPS4B.

Immunofluorescence staining. Huh-7.5 cells cultured on glass coverslips were incubated with LysoTracker™ Deep Red (Invitrogen, DND-99) for 2 h at 37°C. The cells were fixed with 4% paraformaldehyde at room temperature (RT) for 15 min. After being washed with phosphate-buffered saline (PBS) (Nissui Pharmaceutical Co., Ltd., 05913), the cells were permeabilized for 15 min at RT with PBS containing 0.1% Triton X-100 and incubated in PBS containing 1% bovine serum albumin (BSA) (Nacalai Tesque, 01859-47) to block nonspecific reaction for 60 min. The cells were incubated with 1% BSA in PBS containing mouse anti-DGAT1 antibody and rabbit anti-NS5A antibody at

RT for 60 min. The cells were washed three times with PBS and incubated with 1% BSA in PBS containing Alexa Fluor™ 488-conjugated anti-mouse IgG (Invitrogen, A11001), Alexa Fluor™ 488-conjugated anti-rabbit IgG (Invitrogen, A11008), Alexa Fluor™ 594-conjugated anti-rabbit IgG (Invitrogen, A11012), or Alexa Fluor™ 405-conjugated anti-mouse IgG (Invitrogen, A31553) at RT for 60 min. The cells were washed four times with PBS, mounted on glass slides, and examined with a confocal microscope (LSM 700) (Zeiss, Germany).

Proximity ligation assay (PLA). *In situ* PLA was performed using a Duolink In Situ PLA kit (Sigma-Aldrich, DUO92008) as described [33]. Briefly, Huh-7.5 cells were transfected with plasmid pEF1A-NS5A-Myc-His_6 together with pCAG-FLAG-DGAT1 and cultured. At 48 h after transfection, cells grown on poly-D-lysine-coated glass coverslips were fixed with 4% paraformaldehyde for 15 min at RT and then permeabilized with PBS containing 0.1% Triton X-100 for 15 min at RT.

The coverslips were incubated with anti-c-Myc mouse mAb and anti-FLAG rabbit pAb. The samples were washed three times with the wash buffer from the kit. The PLA probes anti-Mouse PLUS (Sigma-Aldrich, DUO92001) and anti-Rabbit MINUS (Sigma-Aldrich, DUO92005), were diluted in the antibody diluent provided with the kit. The samples were incubated for 1 h at 37°C in a humidity chamber. The samples were washed and processed according to the manufacturer's instructions for probe ligation, signal amplification, and mounting. The samples were examined with a confocal microscope (LSM 700) (Zeiss, Germany).

Real-time quantitative reverse transcription-PCR (RT-PCR). The total cellular RNA was isolated using a ReliaPrep RNA cell miniprep system (Promega, Z6012) according to the manufacturer's instructions. The cDNA was generated using a GoScript reverse transcription system (Promega, A5001). The real-time quantitative RT-PCR was performed using TB Green Premix Ex Taq II (Tli RNaseH Plus) (Takara Bio, RR820A) with SYBR green chemistry on the StepOnePlus real-time PCR system (Applied Biosystems, USA) as described previously [24]. The primer sequences were as follows: DGAT1, 5'-GGACTACTCACGCATCATCG-3' and 5'-GGTCTCCAAACTGCATGAGC-3'. As an internal control, human GAPDH gene expression levels were measured using the primer 5'-GCCATCAATGACCCCTTCATT-3' and 5'-TCTCGCTCCTGGAAGATGG-3'.

Acknowledgements

We are grateful to Dr C. M. Rice (The Rockefeller University, New York, NY) for providing us with Huh-7.5 cells and pFL-J6/JFH1 plasmid. We thank Y. Kozaki for the secretarial work.

Data and materials availability

All data are available in the main text.

Disclosure statement

The authors declare that there are no conflicts of interest.

Funding information

This research was supported by grants for Basic and Clinical Research on Hepatitis from the Japan Agency for Medical Research and Development (AMED), nos. JP21fk0210090, JP21fk0210053, and JP21fk0310104; and a grant from the KAKENHI, no. 20K07514. P.Y. was supported by the Program for the Nurturing of Next Generation Leaders Guiding Medical Innovation in Asia of the Ministry of Education, Culture, Sports, Science, and Technology (MEXT) of Japan. This work was also supported in part by grants-in-aid from the Ministry of Health, Labor, and Welfare and MEXT.

ORCID

Putu Yuliandari https://orcid.org/0000-0002-7322-0915

Chieko Matsui https://orcid.org/0000-0003-3428-5334

Lin Deng https://orcid.org/0000-0002-5993-6597

Takayuki Abe https://orcid.org/0000-0003-4184-0527

Hiroyuki Mori https://orcid.org/0000-0002-2390-4016

Shuhei Taguwa https://orcid.org/0000-0001-6728-5651

Chikako Ono https://orcid.org/0000-0002-3965-6715

Takasuke Fukuhara https://orcid.org/0000-0001-5471-8331

Yoshiharu Matsuura https://orcid.org/0000-0001-9091-8285

Ikuo Shoji https://orcid.org/0000-0002-0730-4379

FIGURE LEGENDS

Figure 1. NS5A protein interacts with DGAT1 protein in Huh-7.5 cells. (**A**) Huh-7.5 cells were transfected with either pCAG-FLAG-DGAT1 or empty plasmid together with pEF1A-NS5A-Myc-His_6 as indicated, and cultured. At 48 h after transfection, cells were harvested. Cell lysates were assayed for immunoprecipitation with mouse anti-FLAG mAb, followed by immunoblotting with mouse anti-FLAG mAb (3rd panel) or mouse anti-c-myc mAb (4th panel). The western blots are representative of three independent experiments. *: IgG heavy chain. (**B**) Huh-7.5 cells were plated and cultured for 12 h. Cells were infected with HCV J6/JFH1 at a multiplicity of infection (MOI) of 2. At 5 days post-infection, the cells were stained with anti-DGAT1 mAb followed by Alexa Fluor 488-conjugated goat anti-mouse IgG (*green*) and anti-NS5A pAb followed by Alexa Fluor 594-conjugated goat anti-rabbit IgG (*red*). The cells were stained with Hoechst 33342 for the nuclei (*blue*). The stained cells were examined by scanning laser confocal microscopy and image software. The images are representative of three independent experiments. (**C**) Huh-7.5 cells were transfected with pCAG-FLAG-DGAT1 together with pEF1A-NS5A-Myc-His_6 as indicated, and cultured. At 48 h after transfection, the cells were stained with anti-DGAT1 mAb followed by Alexa Fluor 488-conjugated goat anti-mouse IgG (*green*) and anti-NS5A pAb followed by Alexa Fluor 594-conjugated goat anti-rabbit IgG (*red*). The images are representative of three independent experiments. (**D**) Huh-7.5 cells were transfected with pEF1A-NS5A-Myc-His_6 together with pCAG-FLAG-DGAT1, and cultured. At 72 h after transfection, the cells were stained with anti-DDDDK rabbit pAb and anti-c-myc mouse mAb followed by

a proximity ligation assay (Duolink). The images are representative of three independent experiments.

Figure 2. Mapping of the DGAT1-binding domain on NS5A protein and the NS5A-binding domain on DGAT1 protein. (**A**) Schematic representation of the NS5A protein. AH: amphipathic helix, LCS: low-complexity sequences. Each NS5A deletion mutants (a–h) contains an HA-tag in the N-terminal region. (**B**) Huh-7.5 cells were plated and cultured for 12 h. Cells were transfected with pCAG-FLAG-DGAT1 together with each HA-NS5A mutant plasmid as indicated (Fig. 2A, a–h). At 48 h post-transfection, cells were harvested and cell lysates were immunoprecipitated with anti-FLAG beads. Input samples and immunoprecipitated samples were analyzed by immunoblotting with anti-HA rabbit pAb (*upper panel*), or anti-DDDDK mouse mAb (*lower panel*). The western blots are representative of three independent experiments. IP: immunoprecipitation, IB: immunoblotting. *: IgG light chain. (**C**) Schematic representation of the DGAT1 protein. Each DGAT1 deletion mutant (a–i) contains a FLAG-tag in the N-terminal region. AH: amphipathic helix, EL: extracellular lumen, IL: intracellular lumen, TM: transmembrane. (**D, E, F**) Huh-7.5 cells were plated and cultured for 12 h. Cells were transfected with pCAG-HA-NS5A together with each DGAT1 mutant plasmid as indicated (Fig. 2C, a–i). At 48 h post-transfection, cells were harvested and cell lysates were immunoprecipitated with protein A Sepharose beads. Input samples and immunoprecipitated samples were analyzed by immunoblotting with anti-DDDDK mouse mAb (*upper panel*) or anti-HA rabbit pAb (*lower panel*). The western blots are representative of three independent experiments.

Figure 3. HCV infection induces the lysosomal degradation of DGAT1 protein. (**A**) Huh-7.5 cells were infected with HCV J6/JFH1 at an MOI of 2. Cells were cultured and harvested at 2, 4, and 6 days post-infection. Cells were analyzed by immunoblotting with anti-DGAT1, anti-NS5A, and anti-β-actin mAbs. The level of β-actin served as a loading control. The relative levels of the proteins were quantified by densitometry and are indicated below the respective lanes. Mock cells served as negative controls. (**B**) Huh-7.5 cells were infected with HCV J6/JFH1 at an MOI of 2. Cells were cultured and harvested at the indicated times. Total cellular RNA was extracted, and the levels of DGAT1 mRNA were quantified by the real-time quantitative RT-PCR. The amounts of DGAT1 mRNA were normalized to the amounts of GAPDH mRNA. Mock cells served as negative controls. Data represent the mean ± SEM obtained from three independent experiments performed in triplicate. (**C**) Huh-7.5 cells were infected with HCV J6/JFH1 at an MOI of 2. At 3 days post-infection, 25 μM MG132 was administered to the cells. Cells were cultured for 3, 6, and 9 h, harvested, and analyzed by immunoblotting as indicated. The level of β-actin served as a loading control. The relative levels of the proteins were quantified by densitometry and are indicated below the respective lanes. DMSO: dimethyl sulfoxide. (**D**) Huh-7.5 cells were infected with HCV J6/JFH1 at an MOI of 2. At 3 days post-infection, 10 mM NH_4Cl was administered to the cells. Cells were cultured for 12 h, harvested, and analyzed by immunoblotting as indicated. The relative levels of the proteins were quantified by densitometry and are indicated below the respective lanes. (**E**) Huh-7.5 cells were infected with HCV J6/JFH1 at an MOI of 2. At 4 days post-infection, 2 mM 3-MA was administered to the cells. Cells were harvested, and analyzed by immunoblotting as indicated. The relative levels of the proteins were quantified by densitometry and are indicated below the respective lanes. 3-MA: 3-methyladenine. (**F**)

Huh-7.5 cells were plated and cultured for 12 h. Cells were transfected with increasing amounts of either NS5A plasmid or NS5B plasmid as indicated. At 48 h post-transfection, cells were harvested. Cell lysates were analyzed by immunoblotting with anti-DGAT1 and anti-c-myc mAbs. The relative levels of protein expression were quantitated by densitometry and are indicated below the respective lanes. (A, C-F) The western blots are representative of three independent experiments. The densitometry data corresponds to the representative blot.

Figure 4. DGAT1 protein interacts with HSC70 via the KFERQ motif. (**A**) Schematic representation of DGAT1 protein and its amino acid sequences. The KFERQ motif resides in the region ranging from aa 149 to aa 153 of DGAT1 protein. An expression plasmid for FLAG-DGAT1 Q149A was constructed. (**B**) Huh-7.5 cells were plated and cultured for 12 h. Cells were transfected with pACG-FLAG-DGAT1 or pCAG-FLAG-DGAT1 Q149A. At 48 h post-transfection, cells were harvested and cell lysates were immunoprecipitated with anti-HSC70 mouse mAb. Input samples and immunoprecipitated samples were analyzed by immunoblotting with anti-HSC70 mouse mAb and anti-FLAG mouse mAb. The western blots are representative of three independent experiments.

Figure 5. HCV NS5A is colocalized with DGAT1 in the late endosome and the lysosome. (**A**) Huh-7.5 cells were infected with HCV J6/JFH1 at an MOI of 2. At 4 days post infection, 10 mM NH_4Cl was administered to the cells for 12 hours. The cells were stained with anti-DGAT1 mAb followed by Alexa Fluor 405-conjugated goat anti-mouse IgG (*blue*), anti-NS5A pAb followed by Alexa Fluor 488-conjugated goat anti-rabbit IgG

(*green*), and LysoTracker (*red*). The stained cells were examined using a Zeiss LSM 700 scanning laser confocal microscope and image software. Scale bars, 10 μm. (**B**) Huh-7.5 cells were infected with HCV J6/JFH1 at an MOI of 2. At 3 days post infection, cells were transfected with pDsRed-Monomer-Rab7-C3 plasmid (*red*). At 12 h post-transfection, 10 mM NH_4Cl was administered to the cells for 12 hours. The cells were stained with anti-DGAT1 mAb followed by Alexa Fluor 405-conjugated goat anti-mouse IgG (*blue*), anti-NS5A pAb followed by Alexa Fluor 488-conjugated goat anti-rabbit IgG (*green*). The stained cells were examined using a Zeiss LSM 700 scanning laser confocal microscope and image software. Scale bars, 10 μm. (**C**) Huh-7.5 cells were infected with HCV J6/JFH1 at an MOI of 2. At 3 days post infection, cells were transfected with pLEF-GFP-LC3 (*green*). At 12 h post-transfection, 10 mM NH_4Cl was administered to the cells for 12 hours. The cells were stained with anti-DGAT1 mAb followed by Alexa Fluor 405-conjugated goat anti-mouse IgG (*blue*), anti-NS5A pAb followed by Alexa Fluor 594-conjugated goat anti-rabbit IgG (*red*). The stained cells were examined using a Zeiss LSM 700 scanning laser confocal microscope and image software. Scale bars, 10 μm. The images are representative of three independent experiments.

Figure 6. The HCV infection-induced reduction of DGAT1 is restored in VPS4B knockdown cells. (**A**) shControl and shLAMP2A Huh-7.5 cells were infected with HCV J6/JFH1 at an MOI of 2. At 4 days post-infection, the cells were harvested and the cell lysates were analyzed by immunoblotting with anti-DGAT1 mAb, anti-LAMP-2A pAb, anti-NS5A pAb, and anti-β-actin mAb. The level of β-actin served as a loading control. The relative levels of the proteins were quantified by densitometry and are indicated below the respective lanes. Mock cells served as negative control. (**B**) shControl,

shVPS4A, and shVPS4B Huh-7.5 cells were infected with HCV J6/JFH1 at an MOI of 2. At 4 days post-infection, the cells were harvested, and the cells lysates were analyzed by immunoblotting with anti-DGAT1 mAb, anti-VPS4A pAb, anti-VPS4B mAb, anti-NS5A pAb, and anti-β-actin mAb. The level of β-actin served as a loading control. The relative levels of the proteins were quantified by densitometry and are indicated below the respective lanes. Mock cells served as a negative control. The western blots are representative of three independent experiments. The densitometry data corresponds to the representative blot.

Figure 7. A proposed mechanism of the HCV NS5A-induced lysosomal degradation of DGAT1 via eMI. HCV NS5A interacts with HSC70 and recruits HSC70 to DGAT1 protein through an interaction between NS5A and DGAT1. HSC70 interacts with DGAT1 at its KFERQ motif. Protein complexes are delivered to the late endosomes (MVB) by HSC70 and bind to phosphatidylserine (PS) of the endosomal membrane. HSC70 is internalized along with DGAT1 into microvesicles via the coordinating function of ESCRT I (TSG101), ESCRT III (VPS4B), and accessory protein (Alix). The degradation of microvesicles occurs in the lysosome via endosome-lysosome fusion. ESCRT: endosomal sorting complex required for transport, MVB: multivesicular bodies. NS5A interacts with HSC70 and HNF-1α. HSC70 is recognized by LAMP-2A, a lysosomal membrane protein. HNF-1α is degraded via CMA.

REFERENCES

1. Organization WH. Global progress report on HIV, viral hepatitis, and sexually transmitted infections. WHO Press; 2021.
2. Dubuisson J. Hepatitis C virus proteins. World J Gastroenterol. 2007 May 7;13(17):2406-15.
3. Suzuki R, Suzuki T, Ishii K, et al. Processing and functions of Hepatitis C virus proteins. Intervirology. 1999;42(2-3):145-52.
4. Wakita T, Pietschmann T, Kato T, et al. Production of infectious hepatitis C virus in tissue culture from a cloned viral genome. Nat Med. 2005 Jul;11(7):791-6.
5. Lindenbach BD, Evans MJ, Syder AJ, et al. Complete replication of hepatitis C virus in cell culture. Science. 2005 Jul 22;309(5734):623-6.
6. Sarrazin C. Treatment failure with DAA therapy: Importance of resistance. J Hepatol. 2021 Jun;74(6):1472-1482.
7. Matsui C, Deng L, Minami N, et al. Hepatitis C Virus NS5A Protein Promotes the Lysosomal Degradation of Hepatocyte Nuclear Factor 1alpha via Chaperone-Mediated Autophagy. J Virol. 2018 Jul 1;92(13).
8. Das G, Shravage BV, Baehrecke EH. Regulation and function of autophagy during cell survival and cell death. Cold Spring Harb Perspect Biol. 2012 Jun 1;4(6).
9. Cuervo AM. Autophagy: in sickness and in health. Trends Cell Biol. 2004 Feb;14(2):70-7.
10. Glick D, Barth S, Macleod KF. Autophagy: cellular and molecular mechanisms. J Pathol. 2010 May;221(1):3-12.

11. Feng Y, He D, Yao Z, et al. The machinery of macroautophagy. Cell Res. 2014 Jan;24(1):24-41.

12. Evans CG, Chang L, Gestwicki JE. Heat shock protein 70 (hsp70) as an emerging drug target. J Med Chem. 2010 Jun 24;53(12):4585-602.

13. Kaushik S, Cuervo AM. Chaperone-mediated autophagy: a unique way to enter the lysosome world. Trends Cell Biol. 2012 Aug;22(8):407-17.

14. Matsui C, Yuliandari P, Deng L, et al. The Role of Chaperone-Mediated Autophagy in Hepatitis C Virus-Induced Pathogenesis. Front Cell Infect Microbiol. 2021;11:796664.

15. Wing SS, Chiang HL, Goldberg AL, et al. Proteins containing peptide sequences related to Lys-Phe-Glu-Arg-Gln are selectively depleted in liver and heart, but not skeletal muscle, of fasted rats. Biochem J. 1991 Apr 1;275 (Pt 1):165-9.

16. Chiang HL, Terlecky SR, Plant CP, et al. A role for a 70-kilodalton heat shock protein in lysosomal degradation of intracellular proteins. Science. 1989 Oct 20;246(4928):382-5.

17. Kaushik S, Cuervo AM. The coming of age of chaperone-mediated autophagy. Nat Rev Mol Cell Biol. 2018 Jun;19(6):365-381.

18. Kirchner P, Bourdenx M, Madrigal-Matute J, et al. Proteome-wide analysis of chaperone-mediated autophagy targeting motifs. PLoS Biol. 2019 May;17(5):e3000301.

19. Klionsky DJ, Abdel-Aziz AK, Abdelfatah S, et al. Guidelines for the use and interpretation of assays for monitoring autophagy (4th edition)(1). Autophagy. 2021 Jan;17(1):1-382.

20. Sahu R, Kaushik S, Clement CC, et al. Microautophagy of cytosolic proteins by late endosomes. Dev Cell. 2011 Jan 18;20(1):131-9.

21. Tekirdag K, Cuervo AM. Chaperone-mediated autophagy and endosomal microautophagy: Joint by a chaperone. J Biol Chem. 2018 Apr 13;293(15):5414-5424.

22. Herker E, Harris C, Hernandez C, et al. Efficient hepatitis C virus particle formation requires diacylglycerol acyltransferase-1. Nat Med. 2010 Nov;16(11):1295-8.

23. Camus G, Herker E, Modi AA, et al. Diacylglycerol acyltransferase-1 localizes hepatitis C virus NS5A protein to lipid droplets and enhances NS5A interaction with the viral capsid core. J Biol Chem. 2013 Apr 5;288(14):9915-9923.

24. Matsui C, Shoji I, Kaneda S, et al. Hepatitis C virus infection suppresses GLUT2 gene expression via downregulation of hepatocyte nuclear factor 1alpha. J Virol. 2012 Dec;86(23):12903-11.

25. Matsui C, Rosalyn Sianipar I, Minami N, et al. A single-amino-acid mutation in hepatitis C virus NS5A disrupts physical and functional interaction with the transcription factor HNF-1alpha. J Gen Virol. 2015 Aug;96(8):2200-2205.

26. Tellinghuisen TL, Marcotrigiano J, Gorbalenya AE, et al. The NS5A protein of hepatitis C virus is a zinc metalloprotein. J Biol Chem. 2004 Nov 19;279(47):48576-87.

27. Tellinghuisen TL, Marcotrigiano J, Rice CM. Structure of the zinc-binding domain of an essential component of the hepatitis C virus replicase. Nature. 2005 May 19;435(7040):374-9.

28. Wang L, Qian H, Nian Y, et al. Structure and mechanism of human diacylglycerol O-acyltransferase 1. Nature. 2020 May;581(7808):329-332.

29. Chang S, Sung PS, Lee J, et al. Prolonged silencing of diacylglycerol acyltransferase-1 induces a dedifferentiated phenotype in human liver cells. J Cell Mol Med. 2016 Jan;20(1):38-47.

30. Blight KJ, McKeating JA, Rice CM. Highly permissive cell lines for subgenomic and genomic hepatitis C virus RNA replication. J Virol. 2002 Dec;76(24):13001-14.

31. Bungyoku Y, Shoji I, Makine T, et al. Efficient production of infectious hepatitis C virus with adaptive mutations in cultured hepatoma cells. J Gen Virol. 2009 Jul;90(Pt 7):1681-1691.

32. Bawono RG, Abe T, Shibata Y, et al. NS5A-ISGylation via Lysine 26 Has a Critical Role for Efficient Propagation of Hepatitis C Virus Genotype 2a. Kobe J Med Sci. 2021 Sep 30;67(2):E38-E47.

33. Sianipar IR, Matsui C, Minami N, et al. Physical and functional interaction between hepatitis C virus NS5A protein and ovarian tumor protein deubiquitinase 7B. Microbiol Immunol. 2015 Aug;59(8):466-76.

34. Okamoto T, Nishimura Y, Ichimura T, et al. Hepatitis C virus RNA replication is regulated by FKBP8 and Hsp90. EMBO J. 2006 Oct 18;25(20):5015-25.

35. Fauzyah Y, Ono C, Torii S, et al. Ponesimod suppresses hepatitis B virus infection by inhibiting endosome maturation. Antiviral Res. 2021 Feb;186:104999.

36. Taguwa S, Kambara H, Fujita N, et al. Dysfunction of autophagy participates in vacuole formation and cell death in cells replicating hepatitis C virus. J Virol. 2011 Dec;85(24):13185-94.

37. Deng L, Adachi T, Kitayama K, et al. Hepatitis C virus infection induces apoptosis through a Bax-triggered, mitochondrion-mediated, caspase 3-dependent pathway. J Virol. 2008 Nov;82(21):10375-85.

38. Shirakura M, Murakami K, Ichimura T, et al. E6AP ubiquitin ligase mediates ubiquitylation and degradation of hepatitis C virus core protein. J Virol. 2007 Feb;81(3):1174-85.

39. Deng L, Liang Y, Ariffianto A, et al. Hepatitis C Virus-Induced ROS/JNK Signaling Pathway Activates the E3 Ubiquitin Ligase Itch to Promote the Release of HCV Particles via Polyubiquitylation of VPS4A. J Virol. 2022 Mar 23;96(6):e0181121.
